# Optimized Optical and Thermal Properties of Al-Pigmented Low-Emissivity Coatings by CuCr_2_O_4_ Powder

**DOI:** 10.3390/ma18204717

**Published:** 2025-10-15

**Authors:** Xiaodong Ma, Xiaolong Weng, Biao Wei, Min Zhang, Lun Qi, Yaqin Wang, Le Yuan, Xiaolong Qing, Wei Luo

**Affiliations:** 1 School of Electronic Science and Engineering, University of Electronic Science and Technology of China, Chengdu 610054, China; maxiaodong0625@163.com (X.M.); wengxl59@163.com (X.W.); xiaolong.qing@std.uestc.edu.cn (X.Q.); 2 Inner Mongolia First Machinery Group Co., Ltd., Xi’an Center, Xi’an 710061, China; weibiao95@163.com; 3 National Key Laboratory of Electromagnetic Information Control and Effects, Shenyang 110035, China; uestc_zhangmin@163.com; 4 Key Laboratory of Fluid and Power Machinery, School of Materials Science and Engineering, Xihua University, Chengdu 610039, China; lun_qi163@163.com (L.Q.); wangyqyyxf@sina.com (Y.W.); yuanle.cn@gmail.com (L.Y.)

**Keywords:** low emissivity, low lightness, high temperature resistance, Al/CuCr_2_O_4_

## Abstract

To reduce the lightness and enhance the thermal resistance of Al-pigmented low-emissivity coatings, CuCr_2_O_4_ pigment was introduced into the coating system via ball milling. The results revealed that both ball milling time and Al: CuCr_2_O_4_ mass ratio significantly affect the optical and infrared properties of the coatings. When the milling time reached 9 h, the pigment attained an optimal flake morphology, leading to the best infrared performance of the composite coating. Additionally, the CuCr_2_O_4_ content effectively suppressed the lightness of Al-pigmented coatings. Compared to Al-pigmented low-emissivity coatings, the composite coating with an Al:CuCr_2_O_4_ ratio of 10:2 exhibited a reduction in L* value from 90 to 65. Meanwhile, it retained a low average infrared emissivity of 0.42 in the 3–5 μm and 8–14 μm ranges. Moreover, the incorporation of CuCr_2_O_4_ significantly improved the Al-pigmented coating’s thermal resistance from 500 °C to 600 °C. The composite coating maintained a Grade 1 adhesion rating with heat treatment of 600 °C due to a self-healing effect_._ These composite coatings with low emissivity, low lightness, and high-temperature resistance are highly suitable for high-temperature and infrared stealth applications.

## 1. Introduction

Low-infrared-emissivity coatings, as a type of functional coating, have been widely applied in fields such as building energy conservation, thermal protection, and infrared stealth [1,2,3]. These coatings are typically composed of low-emissivity pigments, binders, and color pigments. The flake Al powder is the predominant low-emissivity pigment due to its low cost, low density, and low infrared absorption characteristics [4,5,6]. Meanwhile, organic resin binders are extensively used in the coatings owing to their strong adhesion to substrates and ease of application. However, the high reflectivity of Al in the visible wavelength range results in high lightness (L*), which significantly compromises the visible-light stealth compatibility of camouflage coatings [7]. In addition, the thermal decomposition of resins at elevated temperatures limits the service temperature of organic low-emissivity Al-pigmented coatings [8]. Therefore, current organic low-infrared-emissivity coatings face a triple challenge: how to simultaneously achieve low infrared emissivity, low visible brightness, and high thermal resistance.

To reduce the lightness of coatings, the conventional approach involves incorporating color pigments into the coating. However, most color pigments exhibit strong absorption in the infrared region, which significantly increases the infrared emissivity of the coating. For example, Yuan et al. [9,10] prepared magnetic Al/Fe_3_O_4_ core–shell pigments via chemical precipitation. Although the L* value of the pigment decreased to 62.3, the infrared emissivity increased substantially, reaching up to 0.6. Liu et al. [11,12,13] fabricated composite particles, which reduced the coating’s L* value to 71.9, but the emissivity increased to 0.44. Wu et al. [14] used a flux capping method to modify Al powder to reduce the gloss of the coating, but the infrared emissivity of the coating was greater than 0.6.

To enhance the thermal resistance of coatings, the most commonly used strategies involve incorporating high-temperature-resistant fillers and employing organic resins with superior thermal stability. For example, the addition of diatomite and heat-resistant ceramic powders into Al-pigmented coatings has been proven to improve the thermal resistance of coatings up to 400 °C; however, surface cracking occurs when the temperature exceeds this threshold [15]. Hu et al. [16,17,18,19] investigated the thermal decomposition mechanisms of epoxy- and polysiloxane-based silicone resins, and developed low-emissivity coatings capable of withstanding temperatures up to 600 °C. However, the mechanical properties of these coatings were not further researched. Zhao et al. [20] used polysiloxane resin and flake Al powder as the binder and pigment, respectively, and the prepared coatings were able to maintain morphological integrity after heat treatment at 500 °C. However, the adhesion rating of coatings deteriorated from grade 1 to grade 5 with increasing heat treatment temperature.

Copper chromite black (CuCr_2_O_4_) is a stable black spinel-type inorganic pigment widely used in coatings, ceramics, and plastics due to its excellent thermal stability, chemical resistance, and high hardness [21,22]. In this work, CuCr_2_O_4_ pigment was introduced into the Al-pigmented coating via ball milling to reduce the coating’s lightness and enhance its thermal resistance. First, CuCr_2_O_4_ pigment was mechanically milled with spherical Al powder to form composite pigments, which were then dispersed into a polysiloxane resin matrix to fabricate composite coatings. The effects of pigment morphology and the Al:CuCr_2_O_4_ mass ratio on the composite coating’s optical and thermal properties were systematically investigated. These findings demonstrate the great potential of the composite coatings for applications in high-temperature and infrared stealth environments.

## 2. Experimental and Characterization

### 2.1. Experimental Materials

Al pigments (particle size of approximately 20 μm) were purchased from Guangzhou Xingbailian Co., Ltd., Guangzhou, China. Methyl/phenyl-siloxane (with a methyl/phenyl ratio of 1.0:1.1 and containing the Si−OH functional group) was purchased from Guangzhou Suomo Chemical Technology Co., Ltd., Guangzhou, China. CuCr_2_O_4_ powders were purchased from Hunan Kelai Material Co., Ltd., Changsha, China.

### 2.2. Preparation of Composite Pigments and Coatings

**Figure 1:**
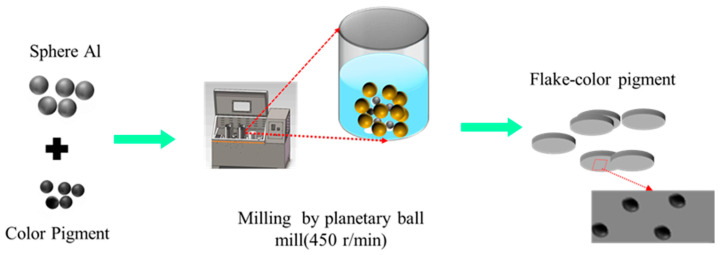
Schematic diagram of the composite pigment preparation process.

Figure 1 presents the preparation process of the composite powder. A total of 10 g spherical Al powder, varying amounts of CuCr_2_O_4_ pigment (0.5 g, 1 g, 1.5 g, and 2 g), 100 g ethyl acetate, and 300 g zirconia milling balls (7 mm in diameter) were added into a planetary ball mill jar. The mixture was milled at a speed of 450 rpm. After milling, the grinding media were removed using a sieve, and the resulting slurry was subjected to vacuum filtration to obtain a filter cake. The filter cake was washed several times to remove residual solvent, then dried in a vacuum oven at 60 °C for 8 h to yield the Al/CuCr_2_O_4_ composite pigment.

The Al/CuCr_2_O_4_ composite pigment was added into polysiloxane resin (solid content of approximately 50 wt.%), and dispersed by mechanical stirring for 120 min. The mixture was then applied to tinplate (12 cm × 5 cm × 0.3 cm) using air spraying. The coatings were dried and fully cured at 200 °C for 2 h. The thickness of the samples was about 40–50 µm, measured using a QuaNix4500 coating thickness gauge (Tianjin QuaNix Electronics Co., Ltd., Tianjin, China).

To study the heat resistance of the samples, the coating specimens were heat-treated in a KSL-1700X sintering furnace (Hefei Kejing Material Technology Co., Ltd., Hefei, China). The temperature was raised from room temperature to the target temperature at a controlled heating rate of 10 °C/min, held at the target temperature for 1 h, and then naturally cooled to room temperature inside the furnace. After heat treatment, the infrared reflectance spectra, surface morphologies, and adhesion of the coatings were characterized.

### 2.3. Characterization and Testing

The structure and phase of Al/CuCr_2_O_4_ particles were analyzed using X-ray diffraction (XRD, SHIMADZU, Kyoto, Japan).

The micro-structure was examined with scanning electron microscopy (SEM, FEI Inspect F50, Hillsboro, OR, USA), equipped with an energy-dispersive X-ray spectrometer (EDX, Hillsboro, OR, USA).

The particle size distribution of pigments was measured by the particle size analyzer (LS-POP(9), Zhuhai, China).

The XPS spectra of samples were characterized by X-ray photoelectron spectroscopy (XPS, Escalab 250Xi, Waltham, MA, USA).

The VIS–NIR reflection spectrum (400–2500 nm) was recorded with FieldSpec 4 Hi-Res NG (Analytical Spectral Devices Inc., Longmont, CO, USA), and the CIE parameters (L*) were determined from the spectrum data.

Infrared reflectance was measured with a Fourier transform infrared spectrometer (FTIR, BRUKER, Tensor27, Karlsruhe, Germany). The average emissivity (ε) of the coating in the 3–5 µm and 8–14 µm ranges was calculated from the infrared reflectance spectrum.

The TMA curve was obtained using a thermal mechanical analyzer (Seiko TMA 6300, Tokyo, Japan) at a heating rate of 10 °C/min. The samples used in the TMA tests were coatings without any heat treatment, and their dimensional changes during heating were recorded.

The cross-cut method was employed to evaluate the coating adhesion. A regular lattice pattern was carefully cut into the coating surface, and a piece of tape was firmly applied over the cut area. The tape was lightly pressed with a fingertip or eraser to enhance its contact with the coating, then quickly peeled off. The adhesion level was determined based on the amount of coating removed.

## 3. Results and Discussion

### 3.1. The XRD Patterns of Composite Powders

**Figure 2:**
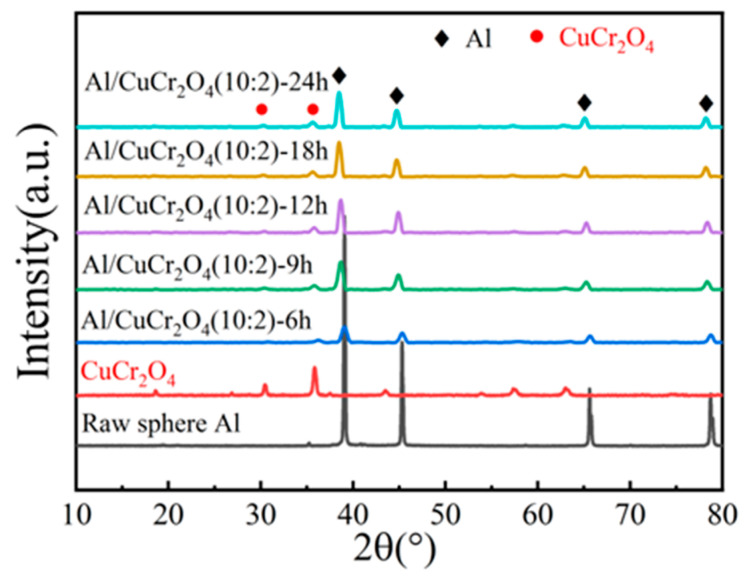
XRD patterns of Al/CuCr_2_O_4_ powders prepared with different ball milling times.

Figure 2 presents the XRD patterns of Al/CuCr_2_O_4_ composite pigments obtained after different milling durations. All the samples exhibit strong diffraction peaks corresponding to the Al phase at 38.47°, 44.8°, 65.1°, and 78.3° (JCPDS No. 85-1327), along with characteristic peaks of CuCr_2_O_4_ at 30° and 35.8°, which correspond to the (112) and (211) crystal planes, respectively (JCPDS No. 87-0432). As the milling time increases from 6 to 24 h, no significant changes are observed in the diffraction peaks of the composite powders, indicating that the pigments solely consist of Al and CuCr_2_O_4_ phases, without secondary phases.

### 3.2. The Morphology and Size Distribution of Composite Powders

**Figure 3:**
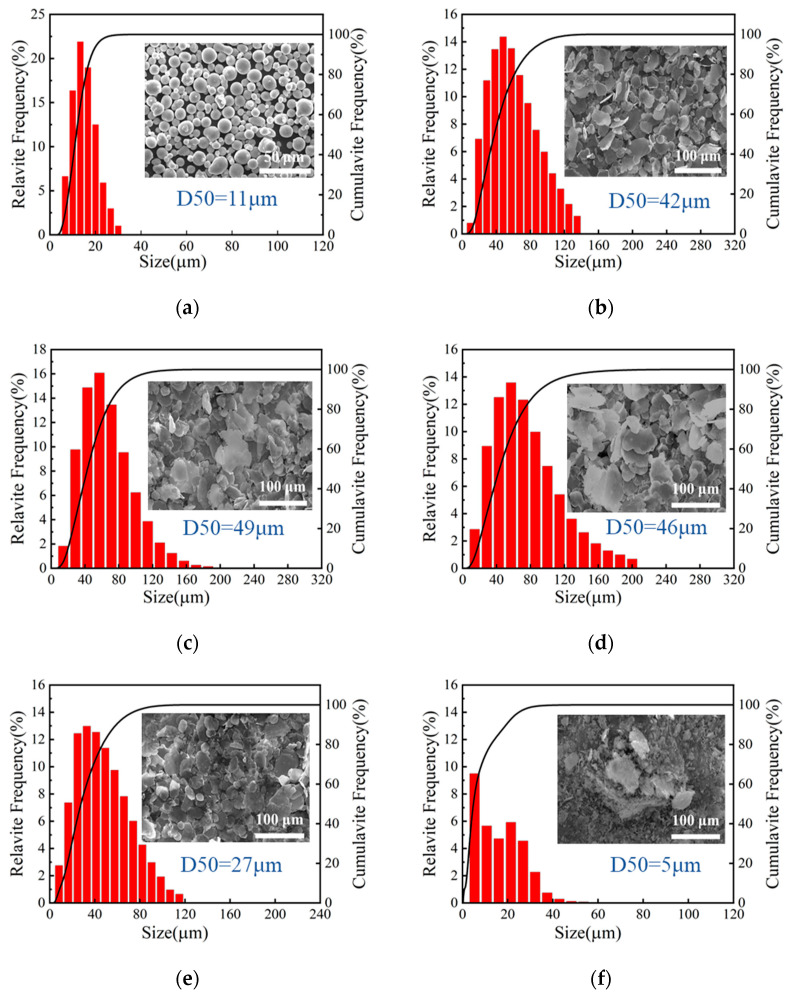
Morphology and particle size distribution of spherical Al powder (**a**) and Al/CuCr_2_O_4_ (10:2) composite powders at different ball milling times: (**b**) 6 h, (**c**) 9 h, (**d**) 12 h, (**e**) 18 h, (**f**) 24 h.

Figure 3 illustrates the particle size distribution and morphological evolution of Al/CuCr_2_O_4_ composite powders prepared with varying milling times. As shown in Figure 3a, the original spherical Al powder exhibits a narrow size distribution primarily in the range from 0 to 40 μm, with a median particle size (D50) of 11 μm. The corresponding SEM image reveals well-defined spherical morphology. After the addition of CuCr_2_O_4_, mechan-ical milling leads to particle flattening and size growth. With milling time increasing from 6 h to 9 h and 12 h (Figure 3b–d), the particles gradually transform from spherical to irreg-ular flaky shapes and the D50 values increase from 42 μm to 49 μm and 46 μm, respec-tively. Upon further milling, the flaky Al structures begin to fracture. At 18 h (Figure 3e), the D50 decreases to 27 μm, and the distribution of particle size narrows. After 24 h of milling (Figure 3f), the flakes are substantially broken, and noticeable agglomeration oc-curs. The size range further narrows to 0–60 μm, with the D50 dropping to 5 μm. Overall, the milling process reveals a distinct morphological evolution from spherical to flattened flakes, followed by fragmentation and agglomeration. Correspondingly, the particle size exhibits a trend of initial increase followed by a decrease with increasing milling time.

### 3.3. The XPS and EDS Analysis of Composite Powders

**Figure 4:**
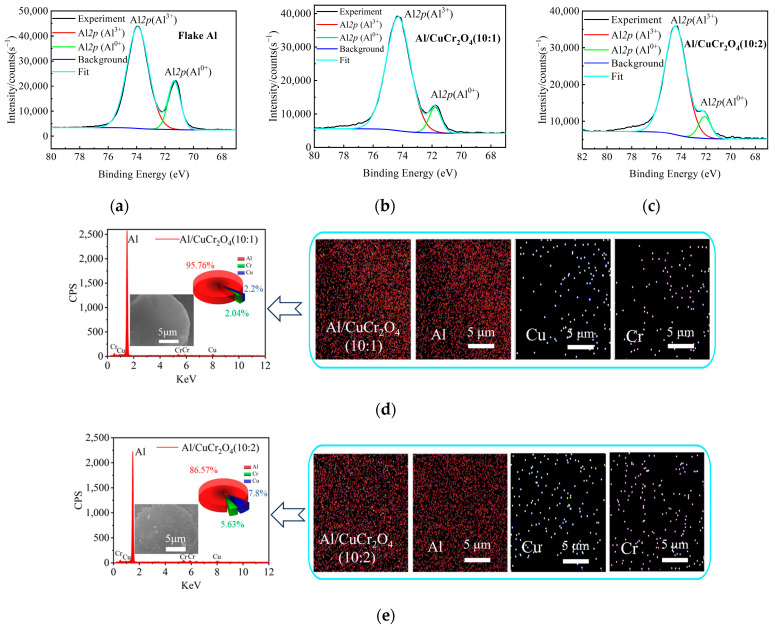
XPS results of powders: (**a**) Flake Al powder, (**b**) Al/CuCr_2_O_4_ (10:1), (**c**) Al/CuCr_2_O_4_ (10:2); EDS analysis of composite pigments: (**d**) Al/CuCr_2_O_4_ (10:1), (**e**) Al/CuCr_2_O_4_ (10:2).

To evaluate the adhesion behavior of CuCr_2_O_4_ pigment on the surface of Al powder, XPS and EDS analyses were conducted. Figure 4a–c show the Al 2p XPS spectra of pure flake Al powder and Al/CuCr_2_O_4_ composite pigments with different mass ratios all after 9 h of ball milling. Each spectrum displays two prominent peaks: the Al^3+^ peak at 74.5 eV corresponds to Al oxide, while the Al^0^ peak at 72 eV represents metallic Al [4]. A comparative analysis reveals that the Al^3+^/Al^0^ peak area ratio is significantly higher in both Al/CuCr_2_O_4_ (10:1) and Al/CuCr_2_O_4_ (10:2) samples than in flake Al powder, with the ratio further increasing in the 10:2 sample. These results indicate that CuCr_2_O_4_ particles have successfully adhered to the surface of the Al, and a higher CuCr_2_O_4_ content leads to a greater degree of surface coverage.

Figure 4d,e present the EDS analysis results and corresponding elemental mapping images of Al/CuCr_2_O_4_ composite pigments with different mixing ratios. The EDS spectra of both two samples clearly exhibit the characteristic Kα peak of Al at 1.49 keV, the Kα (5.41 keV) and Kβ (5.95 keV) peaks of Cr, as well as the Kα (8.04 keV) and Kβ (8.907 keV) peaks of Cu. Notably, no zirconium-related peaks were detected, specifically the Kα at 15.74 keV and Lα at 2.04 keV of Zr [23,24], indicating that the zirconia milling media used during ball milling did not introduce contamination into the composite pigments. The elemental mapping images on the right further confirm the uniform distribution of Al, Cu, and Cr throughout the samples, suggesting that CuCr_2_O_4_ particles are homogeneously deposited on the surface of the flake Al powder, forming a well-dispersed composite structure. Additionally, the pie charts in Figure 4d,e show the elemental composition of the two samples, clearly demonstrating that the relative contents of Cu and Cr increase with higher CuCr_2_O_4_ loading. This further validates the successful preparation of composite pigments with varying ratios.

### 3.4. The Optical and Infrared Properties of Composite Coatings

**Figure 5:**
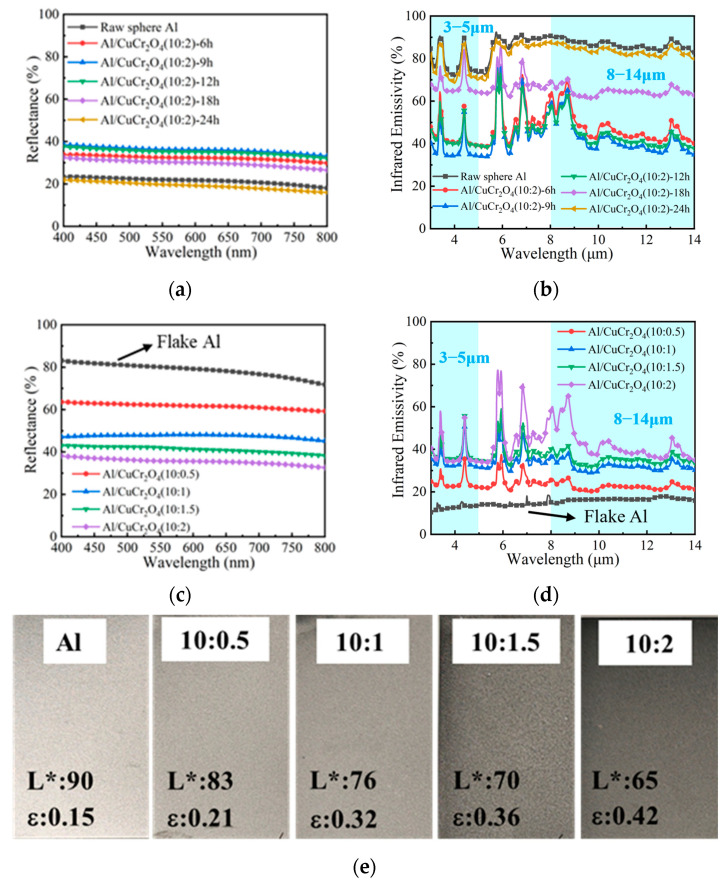
Visible light reflectance (**a**) and infrared emissivity (**b**) of composite coatings at different ball milling times; Visible light reflectance (**c**) and infrared emissivity (**d**) of composite coatings at different Al:CuCr_2_O_4_ mass ratios; (**e**) Photographs of composite coatings at different Al:CuCr_2_O_4_ mass ratios.

To systematically examine the effect of powder morphology on the optical and infrared performance of the coatings, Al/CuCr_2_O_4_ (10:2) composite powders with varying milling times were used as pigments and incorporated into a polysiloxane-based formulation to fabricate composite coatings. Figure 5a,b show the visible reflectance spectra (400–800 nm) and infrared emissivity spectra (3–14 μm) of the composite coatings. The visible reflectance exhibits a trend of initial increase followed by a decrease with increasing milling time which is closely associated with the morphological evolution of the powders during the milling process. In the early stage of ball milling (6–12 h), the spherical particles are gradually compressed into flake-like structures. These flake surfaces enhance specular reflection, resulting in a significant increase in visible reflectance. However, with further milling, the flakes become fragmented into smaller particles. This morphology-driven change in reflectance also directly influences the infrared emissivity of the composite coatings [25]. During the 6–12 h milling stage, the deformation of spherical Al particles into flake-like structures increases the planar surface area and promotes overlapping alignment within the coating, which enhances multiple internal reflections of infrared radiation. This high reflectivity of the flake structures reduces the absorption of infrared radiation, thereby lowering the infrared emissivity. As the milling continues and particle fragmentation progresses, the resulting smaller particle size and irregular surfaces increase both the scattering and absorption of infrared radiation, leading to a rise in emissivity. Notably, the sample prepared after 9 h of ball milling exhibits an optimized flake morphology, resulting in the lowest infrared emissivity.

Based on the composite powders prepared via 9 h of ball milling, a series of coatings with different Al:CuCr_2_O_4_ mass ratios were fabricated to further evaluate their visible and infrared optical properties. As shown in Figure 5c,d, the increase in CuCr_2_O_4_ content led to a gradual decrease in visible reflectance, while the infrared emissivity slightly increased but remained at a relatively low level. Compared with the Al-pigmented coating, the incorporation of CuCr_2_O_4_ significantly reduced the visible reflectance of the composite coatings, which is visually demonstrated in Figure 5e. When the Al: CuCr_2_O_4_ ratio was 10:2, the L* of the coating reduces from 90 to 65, dropping about 28%. Along with the reduction in visible lightness, the average infrared emissivity of composite coating in both the 3–5 μm and 8–14 μm bands remained as low as 0.42, indicating that the composite coating effectively suppresses visible reflectance while maintaining excellent infrared stealth performance.

### 3.5. The Thermal Resistance of Composite Coatings

**Figure 6:**
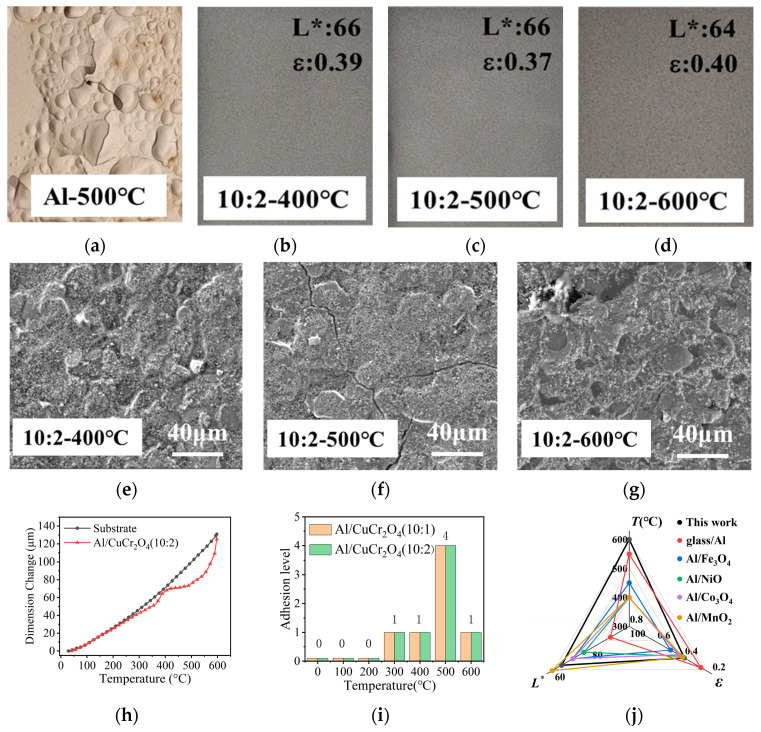
Photographs of coatings after 1 h heat treatment at different temperatures: (**a**) Al-500 °C, (**b**) Al/CuCr_2_O_4_ (10:2)-400 °C, (**c**) Al/CuCr_2_O_4_ (10:2)-500 °C, (**d**) Al/CuCr_2_O_4_ (10:2)-600 °C; SEM surface morphology of composite coatings: (**e**) Al/CuCr_2_O_4_ (10:2)-400 °C, (**f**) Al/CuCr_2_O_4_ (10:2)-500 °C, (**g**) Al/CuCr_2_O_4_ (10:2)-600 °C; (**h**) Dimensional Change of coating and substrate at different temperatures; (**i**) Adhesion level of composite coatings after heat treatment (Grade 0 indicates optimal adhesion, Grade 5 indicates poorest adhesion); (**j**) Performance comparison radar chart for composite coating with five representative works [8,9,11,12,13].

To further evaluate the high-temperature resistance of the composite coatings, coatings (with a fixed Al:CuCr_2_O_4_ mass ratio of 10:2) were subjected to thermal treatments at various temperatures. The corresponding surface morphology, thermal expansion behavior and coating adhesion were analyzed. As shown in Figure 6a–g, photographs and SEM images illustrate the surface conditions of the coatings after 1 h of heat treatment at different temperatures. Figure 6a shows the Al-pigmented coating after treatment at 500 °C, which exhibits severe surface cracking and delamination. In contrast, Figure 6b–d display composite coatings containing Al/CuCr_2_O_4_ pigment, which remain intact across the 400–600 °C range. The SEM images in Figure 6e–g provide further insight into the microstructural evolution. After heat treatment at 400 °C (Figure 6e), the composite coating surface remains flat and compact. When the temperature increases to 500 °C (Figure 6f), pronounced surface cracks begin to form. This phenomenon is primarily due to the depolymerization of the polysiloxane resin, which generates low-molecular-weight cyclic siloxanes and intensifies polymer chain scission [26]. However, after treatment at 600 °C (Figure 6g), the previously formed cracks disappear, and the surface becomes dense again. This unique “self-healing” behavior is attributed to the thermal cleavage and rearrangement of Si-O-Si bonds in the resin backbone [27,28], during which the polysiloxane resin undergoes a scission–rearrangement–repolymerization process at elevated temperatures, ultimately forming a thermally stable inorganic silica network. This phenomenon has been reported in our previous study [20]. Simultaneously, the mobility and diffusion of the low-molecular-weight siloxane species facilitate the filling of surface cracks, resulting in a re-dense and flat coating layer. These results demonstrate that the incorporation of CuCr_2_O_4_ pigment significantly enhances the thermal stability of the Al-pigmented coating. After treatment at 500 °C, only minor surface cracking in the composite coating is observed under a microscope, which facilitates the subsequent self-healing behavior at 600 °C.

To further investigate the thermal resistance properties of Al/CuCr_2_O_4_ composite coating, the dimensional variations between the coating and substrate under different thermal treatment temperatures, as well as the coating adhesion performance, are tested. As shown in Figure 6f, the composite coating and substrate exhibit approximately linear thermal expansion within the temperature range of room temperature (RT) to 400 °C. However, when the temperature reaches 500 °C, a pronounced thermal mismatch occurs between the composite coating and substrate. With the temperature further increasing, their thermal expansions gradually converge. Figure 6g presents the adhesion test results of the composite coatings (Grade 0 indicates optimal adhesion, Grade 5 indicates poorest adhesion [29]). After thermal treatment from room temperature to 400 °C, all the composite coatings maintain excellent adhesion, with ratings of Grade 1 and Grade 0. This indicates that all the composite coatings present good temperature stability within this temperature range. Upon heating to 500 °C, the adhesion strength of all composite coatings significantly deteriorates to Grade 4, primarily attributed to the depolymerization of the polysiloxane matrix at this temperature. This result is consistent with the crack formation observed in the SEM analysis. However, when the temperature increases to 600 °C, the adhesion performance improves substantially, returning to a level of Grade 1. This “self-healing” phenomenon has been observed in the SEM images, which indicates that scission and rearrangement of Si-O-Si bonds not only repair surface cracks, but also significantly enhance the interfacial bonding strength between the coating and the substrate. In Figure 6j, the overall performance of the composite coating is summarized, demonstrating that its lightness (L), infrared emissivity (ε), and thermal resistance (T) are superior to those of materials reported in the previous literature.

**Figure 7:**
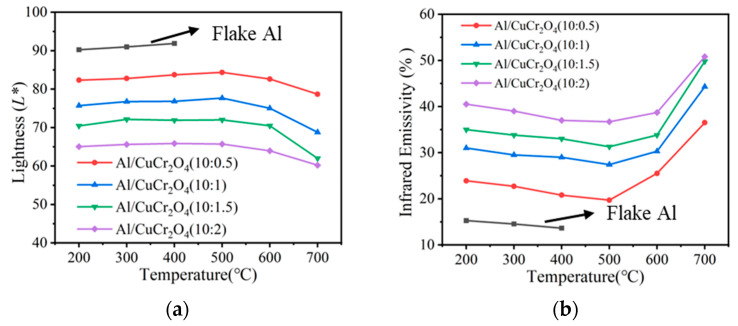
L* value (**a**) and average infrared emissivity (**b**) of coatings after 1 h heat treatment at different temperatures.

Finally, the L* values and average infrared emissivity of the composite coatings after heat treatment at various temperatures were measured and calculated, as shown in Figure 7a,b. Below 600 °C, both the L* values and infrared emissivity of the coatings remained nearly unchanged with increasing temperature. However, when the treatment temperature reached 700 °C, the infrared emissivity significantly increased, which is due to the oxidation of the Al [8]. In summary, compared with Al-pigmented low-emissivity coatings, the composite coatings exhibit superior optical and mechanical stability at temperatures below 600 °C.

## 4. Conclusions

In this work, low-emissivity coatings containing Al/CuCr_2_O_4_ composite pigment were successfully fabricated. At an Al:CuCr_2_O_4_ mass ratio of 10:2, the composite coating exhibited a lightness as low as 65, reduced 28% compared to Al-pigmented low-emissivity coatings with the value of 90. Meanwhile, it maintained a low average infrared emissivity of just 0.42 in both 3–5 μm and 8–14 μm spectral ranges. Moreover, the thermal resistance of the composite coatings up to 600 °C due to the “damage–self-healing” behavior. The properties of low emissivity, low lightness and high-temperature resistance make the composite coatings show great promise in advanced military equipment.

## Data Availability

The data presented in this study are available on request from the corresponding author due to privacy.
